# Modelling Ranavirus Transmission in Populations of Common Frogs (*Rana temporaria*) in the United Kingdom

**DOI:** 10.3390/v11060556

**Published:** 2019-06-15

**Authors:** Amanda L.J. Duffus, Trenton W.J. Garner, Richard A. Nichols, Joshua P. Standridge, Julia E. Earl

**Affiliations:** 1 Department of Mathematics and Natural Sciences, Gordon State College, Barnesville, GA 30204, USA; js197212@gordonstate.edu; 2 Institute of Zoology, Zoological Society of London, London NW1 4RY, UK; trent.garner@ioz.ac.uk; 3 School of Biological and Chemical Sciences, Queen Mary, University of London, London E1 4NS, UK; r.a.nichols@qmul.ac.uk; 4 School of Biological Sciences, Louisiana Tech University, Ruston, LA 71272, USA; jearl@latech.edu

**Keywords:** transmission modelling, susceptible-infected (SI) models, emerging infection, ranavirosis, *Iridoviridae*, disease dynamics

## Abstract

Ranaviruses began emerging in common frogs (*Rana temporaria*) in the United Kingdom in the late 1980s and early 1990s, causing severe disease and declines in the populations of these animals. Herein, we explored the transmission dynamics of the ranavirus(es) present in common frog populations, in the context of a simple susceptible-infected (SI) model, using parameters derived from the literature. We explored the effects of disease-induced population decline on the dynamics of the ranavirus. We then extended the model to consider the infection dynamics in populations exposed to both ulcerative and hemorrhagic forms of the ranaviral disease. The preliminary investigation indicated the important interactions between the forms. When the ulcerative form was present in a population and the hemorrhagic form was later introduced, the hemorrhagic form of the disease needed to be highly contagious, to persist. We highlighted the areas where further research and experimental evidence is needed and hope that these models would act as a guide for further research into the amphibian disease dynamics.

## 1. Introduction

Ranaviruses are double-stranded DNA viruses in the *Iridoviridae* family that can infect ectothermic vertebrates [1] and are found worldwide [2]. Ranaviruses cause systemic hemorrhaging and edema in amphibian, reptile, and fish hosts [3]. In amphibians, mortality can occur in only three days in very susceptible host species and life history stages [4], resulting in die-offs of both adults and larvae, in Europe [5,6], and tadpoles and metamorphs, in North America [7,8]. Ranaviruses have been shown to alter amphibian population dynamics, with declines of the common frog (*Rana temporaria*) in the United Kingdom [9] and whole amphibian communities in Spain [10]. Additionally, population simulation models have shown that ranaviruses could potentially cause the local extinction of populations of three, highly susceptible species of the United States anurans [11,12]. However, these models do not include the transmission dynamics, which are not well-understood and could potentially alter host population dynamics. Farrell et al. [13] modeled host population declines in the case of the highly pathogenic *Ambystoma tigrinum virus* (ATV), but they found that extinction of host populations would not occur even in the cases where the host population was severely reduced. These diverse conclusions showed that there were many different possible outcomes of ranavirus infections that influenced disease dynamics, and they appeared to be system-specific.

Mathematical models are helpful tools for understanding transmission dynamics and the persistence of pathogen and host populations [14,15]. Models might help determine which transmission conditions are most likely, given the information about disease dynamics in wild populations. In the case of the ranavirus, Brunner and Yarber [16] developed a model which suggested that ranavirus transmission from water and scavengers are likely minimal in most circumstances. Previous attempts have also been made to formalize the transmission dynamics at, both, the species level [17] and at the community level [18]. Most models have been based on North American amphibian populations or communities. The dynamics of amphibian–ranavirus systems in the UK, the ranavirus–common frog (*Rana temporaria*) system, are distinctly different from those in North America and thus require specific investigation.

Here, we developed susceptible-infection (SI) models for ranavirus infection in common frogs, in the UK. The ranavirus–common frog system in the UK differs from the infection/disease dynamics seen in North American anuran species, because infections in eggs or tadpoles of wild populations are absent, for the most part [19,20], despite experimental evidence that tadpoles can be infected and have died with signs of ranavirosis. Given this information, the route of transmission in common frogs is most likely among adults. With the benefit of long-term data sets on the persistence of ranavirus infections in common frogs, we know that the ranavirus can persist in adult frog populations for many years [9] and involves at least two distinct disease syndromes (hemorrhagic and ulcerative) with variations in the prevalence of disease, across sites and years [21]. We, therefore, developed SI models to test the hypothesis that ranaviruses have the potential to remain in the UK common frog populations, under various conditions, with only horizontal transmission between adults. We examined scenarios with host population declines that included both ranavirus syndromes.

## 2. Basic Model Formulation

**Figure 1:**
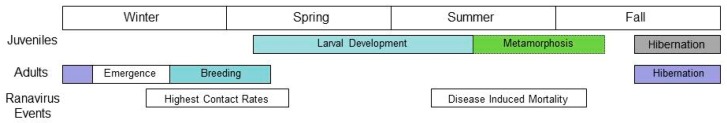
Annual cycle of important life history events for common frogs (*Rana temporaria*) and important events for ranavirus infections and diseases, for these animals. Boxes shaded in blue are those that occur in the aquatic environment, green boxes are those that straddle the land and water, grey boxes occur at an unknown location, and mauve boxes are life history events that are known to happen on both the land and in the water.

In the simplest case, we used an SI model with no recovery, based on the high mortality rate associated with ranavirus infection. We followed the method for developing mathematical models described by Otto and Day [22]. The population consisted of recruits (A_R_), susceptible individuals (A_S_), and infected individuals (A_I_) and mortality occurred at two different rates—natural mortality (M_N_) and mortality due to disease (M_D_). We assumed that the adult population size remained constant (i.e., mortality was fully compensated irrespective of the cause of death), all recruits to the population were susceptible to the ranavirus, and all individuals were equally susceptible to infection. The contact rate (Ψ) was defined as the number of different individuals that one animal physically contacts, the likelihood of transmission (σ) was the probability of transmission given the physical contact, and R_0_ was the basic reproductive rate of the virus. We used a discrete time model, because common frogs aggregate annually for breeding and we assumed that transmission primarily occurred from contacts during breeding. We also assumed that mortality due to disease occurred primarily during summer; ranavirus-associated mortality peaks between mid-July and mid-August, after breeding, has previously been concluded [20]. (See Figure 1 for a schematic view of the important life history events and timing.) Based on the above assumptions, we developed the following equations:

A_s_(t + 1) = A_s_(t) − σΨ·A_s_(t)·A_I_(t) − M_N_(t) + A_R_(t) (1)

A_I_(t + 1) = A_I_(t) + σΨ·A_s_(t)·A_I_(t) − [M_N_(t) + M_D_(t)] (2)

R_0_ = σΨ·A_s_(t)/[M_N_(t) + M_D_(t)] (3)

To determine under what conditions the ranavirus would remain or spread in a population, we modified Equation (3) by removing M_D_ (t) to assume a successful introduction:

R_o_ = σΨ·A_s_/M_N_(t) (4)

**Figure 2:**
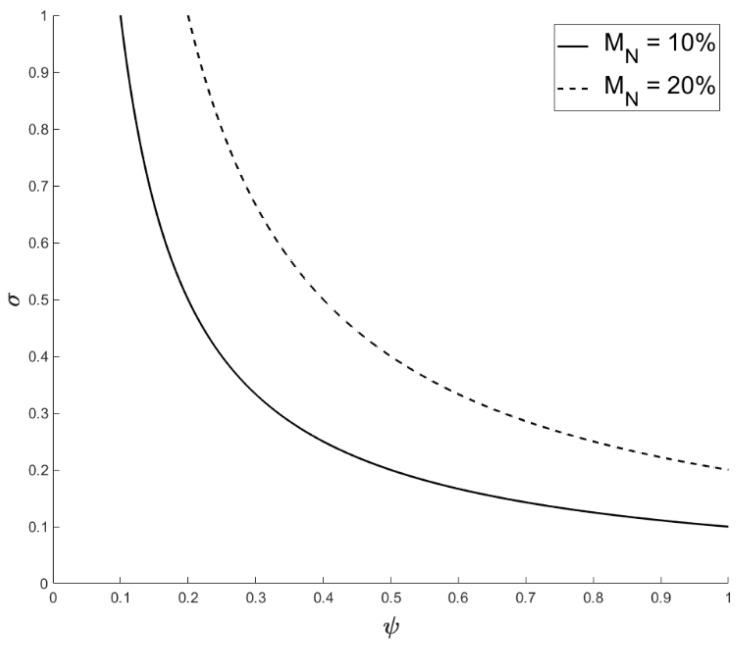
The interaction between σ and Ψ, under which R_o_ ≥ 1, when A_S_ = 99 and an M_N_ = 20% (upper dashed-curve) and when the initial conditions of A_S_ = 49 and an M_N_ = 10% (lower curve), where Ψ is the contact rate and σ is the likelihood of transmission for the model.

The important interactions in this equation are between σ and Ψ, and this relationship needed to be explored, graphically, to determine when the conditions for R_o_ ≥ 1 exist. If we assume a population size of 99 (A_S_), with an initial introduction of one infected individual (A_I_) and an M_N_ of 20% [23] (although other estimates do exist [24]), values of σ and Ψ under which R_o_ ≥ 1 are illustrated in Figure 2. To test if similar assumptions are valid at a smaller population size, we repeated the process using alternative values for A_S_ = 49 and M_N_ = 10% (i.e., 5 individuals/annum) and introduced one infected individual (total population size 50; Figure 2).

After establishing the conditions which permit ranavirus persistence, we next examined the behavior of A_S_ and A_I_ under potential biologically relevant conditions. We used experimental data from ranavirus exposures in the literature, to estimate σ (Table 1). It is important to note that the data used to estimate σ was extremely variable; the viral titers used in the experiments range from TCID_50_ of 10^1^ to 10^2^ mL for virus obtained from crude organ homogenates to TCID_50_ of 10^4.2^ to 10^6.2^ mL, for virus produced via tissue culture [25]. This variability made the biological relevance of our estimates less than ideal; however, these are the best estimates that can be made with the available data.

**Figure 3:**
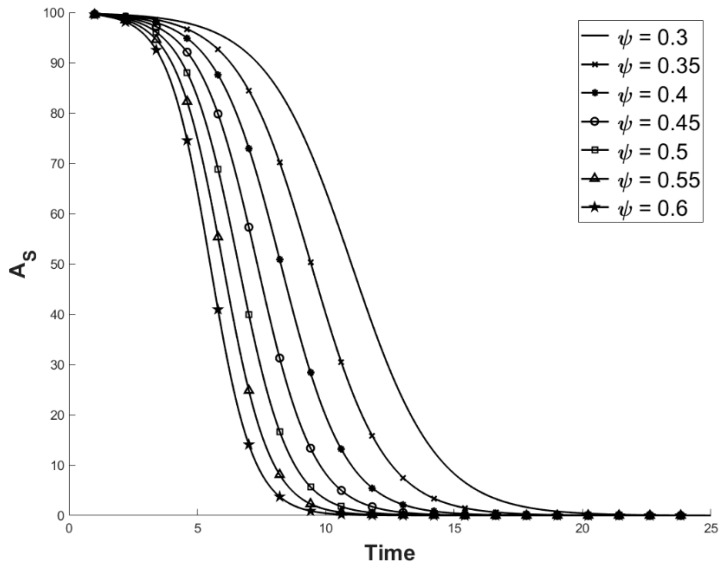
Predicted values of A_S_ with varying values of Ψ while other values remained constant at: σ = 0.3; M_N_ = 0.2; and M_D_ = 0.75. The starting population composition is A_I_ = 1 and A_S_ = 99 and time is in years. A_S_ is the number of susceptible individuals, Ψ is the contact rate, σ is the likelihood of transmission, M_N_ is the natural mortality rate, and M_D_ is the mortality rate associated with ranavirosis.

**Figure 4:**
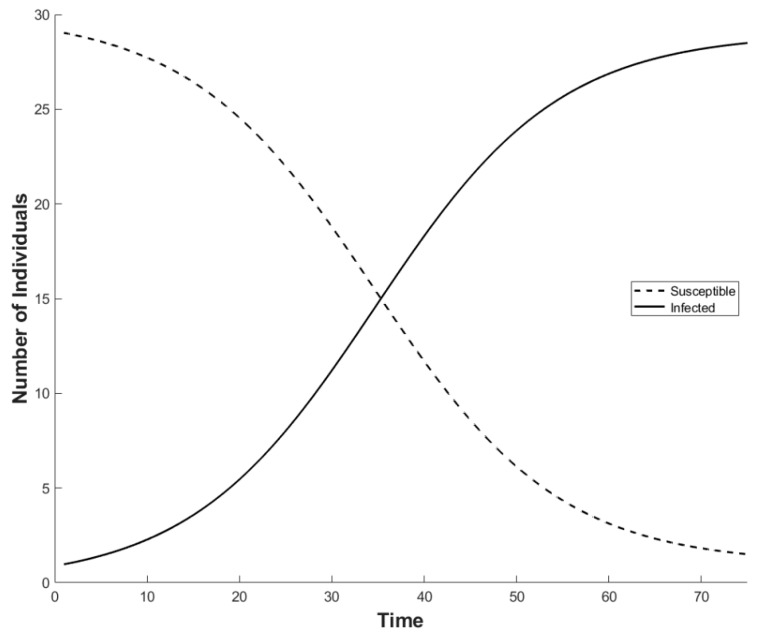
The average expectation of the ranavirus dynamics in a population of adult common frogs (*Rana temporaria*) through time (years). (Ψ = 0.45; σ = 0.3; M_N_ = 0.2; M_D_ = 0.775; starting population comprised of A_I_ = 1 and A_S_ = 29.) A_I_ is the number of infected individuals, A_S_ is the number of susceptible individuals, Ψ is the contact rate, σ is the likelihood of transmission, M_N_ is the natural mortality rate, and M_D_ is the mortality rate associated with ranavirosis.

Estimates of Ψ were unavailable, so we arbitrarily chose Ψ = 0.3→0.6 (Figure 3). We ignored sex specific contact rates, as male–male contact rates are extremely high and polyandry is common [26]. Additionally, Teacher et al. [9] found that the median population size of common frogs was 31 for those places where ranaviruses have emerged in the UK, so we used an A_Total_ of 30 (Figure 4).

**Figure 5:**
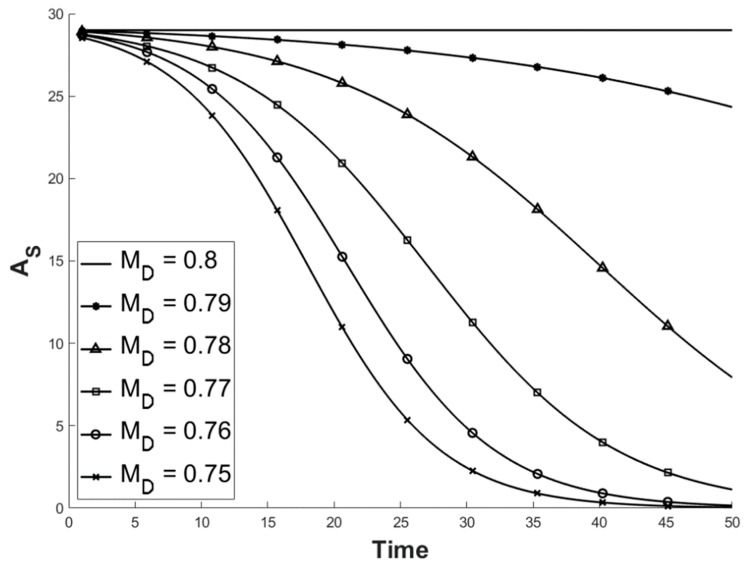
Illustration of the predicted values for A_S_ with different disease-induced mortality rates, while other values remained constant at: Ψ = 0.45; σ = 0.3; M_N_ = 0.2; the starting population comprised of A_I_ = 1 and As = 29. A_s_ is the number of susceptible individuals, Ψ is the contact rate, σ is the likelihood of transmission, M_N_ is the natural mortality rate and M_D_ is the mortality rate associated with ranavirosis.

Since ranaviruses have emerged recently in the UK, and common frogs and the severity of the disease is affected by climate change, it is unlikely that the disease dynamics have reached an equilibrium ([27,28]; Figure 4). Using an estimated σ of 0.3 (from data from Table 1), an average M_D_ of 0.775, an average Ψ value of 0.45, and an A_total_ of 30 [9] with the initial conditions of an A_s_ of 29 and an A_I_ of 1, we found that the interaction between A_S_ and A_I_ requires greater than 60 years to stabilize to post-epidemic dynamics (Figure 5). These results suggest that, under the present conditions, ranavirus in populations of common frogs can be sustained entirely through adult–adult transmission.

### 2.1. Factoring in Population Decline

In UK common frogs, ranavirosis has caused an 81% decline in some affected populations, over a 10 year period [9]; this appears to be a clear violation of the assumption in the model that population size remains constant. Teacher et al. [9] found that the declines in these populations were proportional to their size (i.e., the larger the population, the larger the decline experienced). This is possibly consistent with the assumption that all adults are equally susceptible, when a ranavirus first invades a population. Contrarily, larger declines in larger populations might be due to density-dependent transmission of the virus. This scenario can also account for the differences among individuals, in their susceptibility to the ranavirus.

**Figure 6:**
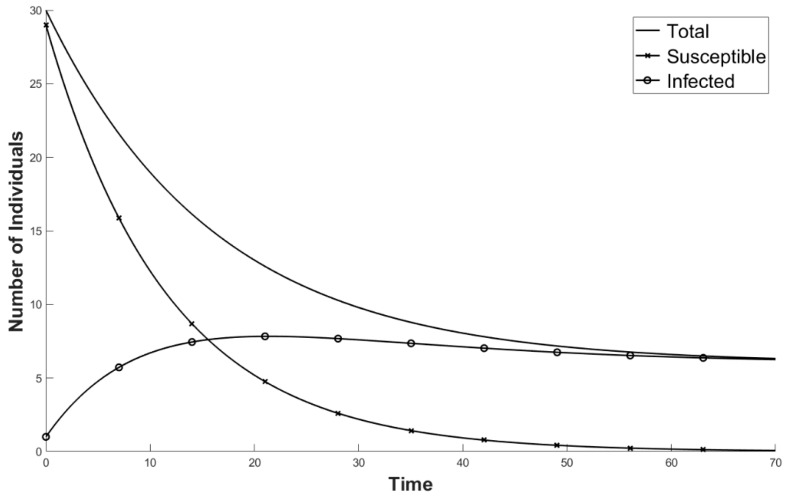
Illustration of the predicted dynamics of a common frog population, with the ranavirus factoring in an annual population decline of 8.1% for adult common frogs. (Ψ = 0.45; σ = 0.3; M_N_ = 0.2; M_D_ = 0.775; starting population comprised of A_I_ = 1 and A_S_ = 29; time is in years.) A_s_ is the number of susceptible individuals, Ψ is the contact rate, σ is the likelihood of transmission, M_N_ is the natural mortality rate, and M_D_ is the mortality rate associated with the ranavirosis.

However, Teacher et al. [29] report that populations of common frogs maintain allelic diversity through the immigration of adults from nearby ponds. This immigration has two potentially important consequences for the emergence of ranavirosis: (1) the population of adult frogs in the affected population will remain susceptible to infection because of the homogenizing effect of immigration; thus, the immigration of susceptible individuals might dilute the prevalence of the resistance genes, as long as the immigrants come from susceptible populations; and (2) immigration might bolster population numbers and reduce the observed decline. Consequently, the estimates of population decline due to ranavirosis that were made by Teacher et al. [9] might, in fact, be underestimates. In addition, Campbell et al. [30] have found that the population structure shifts from older adult frogs to proportionally more juveniles, with a much smaller total population size in the affected populations. These smaller populations have also been shown to be more susceptible to stochastic events, via population modelling [30]. For simplicity, we assumed that declines of 81% over a 10 year period, as seen by Teacher et al. [9], correspond to a steady annual decline of 8.1%. Under such conditions, it takes the model approximately 65 years to stabilize, with all adults in the population suffering from ranavirus infections (Figure 6).

### 2.2. Accounting for Different Disease Syndromes

Ranavirosis in the UK common frogs presents as two syndromes which are not mutually exclusive. The ulcerative form of the disease is characterized by ulcers of the skin and the skeletal muscle, and sometimes necrosis of the digits, while the hemorrhagic form of the disease is characterized by internal hemorrhages, most commonly involving the gastrointestinal and reproductive tracts ([21]; personal observation). Adult common frogs exposed to a tissue homogenate derived from skin ulcers only developed the ulcerative form of the disease (with a prevalence of ~30%; See Table 2; [25]). Conversely, adult frogs exposed to a virus isolate obtained from skin ulcers generated both ulcerative and hemorrhagic signs of the disease, while virus isolated from a hemorrhage caused the hemorrhagic form of the disease in exposed adults [25]. These, and other data, indicate that ranaviruses associated with different pathologies in the UK might have different transmission rates [25]. Our estimates of σ for both the ulcerative and hemorrhagic forms can be found in Table 2. If we take the view that infection using viral isolates derived from the cell culture does not mimic the natural process, the other estimates from Table 2 would be preferred, in which case, we estimated σ for the ulcerative and hemorrhagic syndromes as 0.33 and 0.20, respectively.

**Figure 7:**
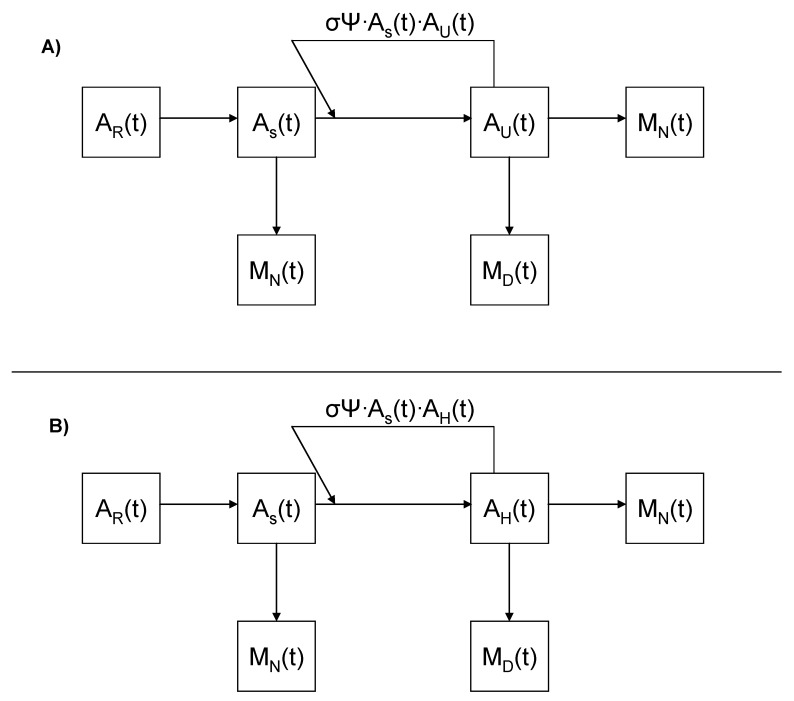
Diagrammatic representations of the transmission dynamics of the ranavirus when only the A_S_ or A_H_ causing isolate of the ranavirus is present. (**A**) When only the ulcerative form of the ranavirus is present within the population. (**B**) When only the hemorrhagic form of the disease is present in the population. All of the variables present are the same as described above and all have a time component associated with them. Where A_s_ is the number of susceptible individuals, Ψ is the contact rate, σ is the likelihood of transmission, M_N_ is the natural mortality rate, and M_D_ is the mortality rate associated with ranavirosis.

**Figure 8:**
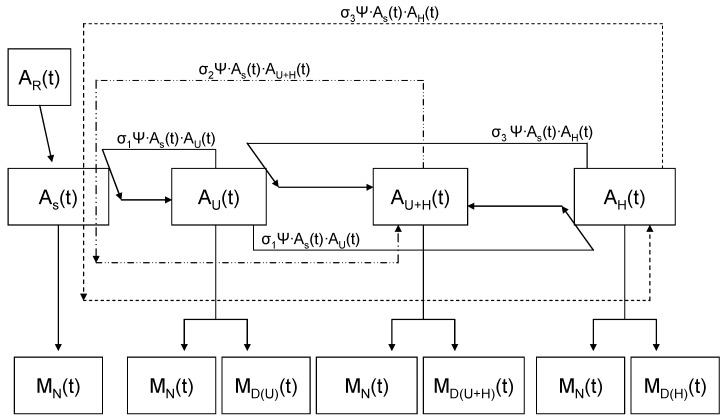
Illustration of the complex transmission dynamics of the ranavirus, when both of the observed disease syndromes are present in the population. Dashed lines are used to make the disease syndrome-specific vectors of the transmission easier to follow. The box sizes are not representative of the number of individuals in each category. The order of the boxes does not indicate when the given disease syndrome was introduced. All parameters have time components associated with them. A_s_ is the number of susceptible individuals, Ψ is the contact rate, σ is the likelihood of transmission, M_N_ is the natural mortality rate, and M_D_ is the mortality rate associated with ranavirosis.

We investigated three scenarios applying different values of σ, where the first two versions explored the two syndromes in isolation (ulcerative form, A_U_, and hemorrhagic form, A_H_, present, respectively, in Figure 7A,B), represented by Equations (5)–(9) for the initial frequencies and Equations (10)–(14) at time (t). Then we developed a new series of equations for when both disease syndromes are present and including individuals that show signs of both forms of the disease, A_U+H_. For simplicity, we assumed that all animals are equally susceptible to both forms of the disease (Figure 8) and that there was no difference in the disease-induced mortality rate between syndromes:

A_S_(t) = A_S_(i) − [σ_1_Ψ·A_S_(i)·A_U_(i) + σ_2_Ψ·A_S_(i)·A_(U+H)_(i) + σ_3_Ψ·A_S_(i)·A_H_(i)] − M_N_(i) + A_R_(i) (5)

A_U_(t) = A_U_(i) + [σ_1_Ψ·A_S_(i)·A_U_(i)] − [σ_1_Ψ·A_U_(i)·A_H_(i) + σ_3_Ψ·A_H_(i)·A_U_(i)] − [M_N_(i) + M_D(U)_(i)] (6)

A_(U+H)_(t) = A_(U+H)_(i) + [σ_2_Ψ·A_S_(i)·A_(U+H)_] + [σ_1_Ψ·A_U_(i)·A_H_(i)] + [σ_3_Ψ·A_H_(i)·A_U_(i)] − [M_N_(i) + M_D(U+H)_(i)] (7)

A_H_(t) = A_H_(i) + [σ_3_β·A_S_(i)·A_H_(i)] − [σ_1_β·A_U_(i)·A_H_(i) + σ_3_β·A_H_(i)·A_U_(i)] − [M_N_(i) + M_D(H)_(i)] (8)

A_s_(t + 1) = A_S_(t) − [σ_1_Ψ·A_S_(t)·A_U_(t) + σ_2_Ψ·A_S_(t)·A_(U+H)_(t) + σ_3_Ψ·A_S_(t)·A_H_(t)] − M_N_(t) + A_R_(t) (9)

A_U_(t + 1) = A_U_(t) + [σ_1_Ψ·A_S_(t)·A_U_(t)] − [σ_1_Ψ·A_U_(t)·A_H_(t) + σ_3_Ψ·A_H_(t)·A_U_(t)] − [M_N_(t) + M_D(U)_(t)] (10)

A_(U+H)_(t + 1) = A_(U+H)_(i) + [σ_2_Ψ·A_S_(i)·A_(U+H)_] + [σ_1_Ψ·A_U_(i)·A_H_(i)] + [σ_3_Ψ·A_H_(i)·A_U_(i)] − [M_N_(i) + M_D(U+H)_(i)] (11)

A_H_(t + 1) = A_H_(t) + [σ_3_Ψ·A_S_(t)·A_H_(t)] − [σ_1_Ψ·A_U_(t)·A_H_(t) + σ_3_Ψ·A_H_(t)·A_U_(t)] − [M_N_(t) + M_D(H)_(t)] (12)

Ulcerative Syndrome

R_oU_ = σ_1_Ψ [A_S_(t) + A_H_(t)]/M_N_(t)+M_D(U)_(t) + M_D(H)_(t) (13)

Hemorrhagic Syndrome

R_oH_ = σ_3_Ψ [A_S_(t) + A_U_(t)]/M_N_(t) + M_D(H)_(t) + M_D(U)_(t) (14)

**Figure 9:**
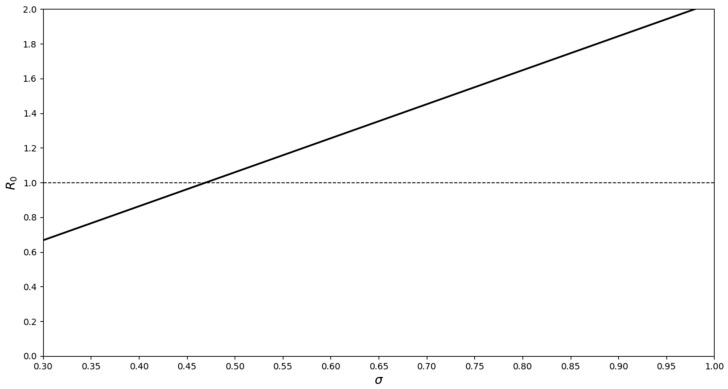
R_o_ values for the introduction of one A_H_ individual into a population of A_S_ = 28 and A_U_ = 1 (M_D(U)_ = M_D(H)_ = 0.775, Ψ = 0.45, M_N_ = 0.2). A_S_ is the number of susceptible individuals, Ψ is the contact rate, σ is the likelihood of transmission, M_N_ is the natural mortality rate, and M_D_ is the mortality rate associated with ranavirosis.

Under the assumptions that A_S_ = 28 → 14, A_H_ = 1 → 14, with the introduction of 1 → 5 A_U_, M_D(U)_ = M_D(H)_ = 0.775, σ_1_ = 0.3, σ_3_ = 0.25, Ψ = 0.45, and M_N_ = 0.2, based on the R_O_ values, A_U_ and A_H_ should not coexist over the long term. It does not matter if A_U_ is introduced into a population with A_H_ or vice-versa (Figure 9). Both of these disease syndromes co-occur in nature [21] and in experimental infections [25].

**Figure 10:**
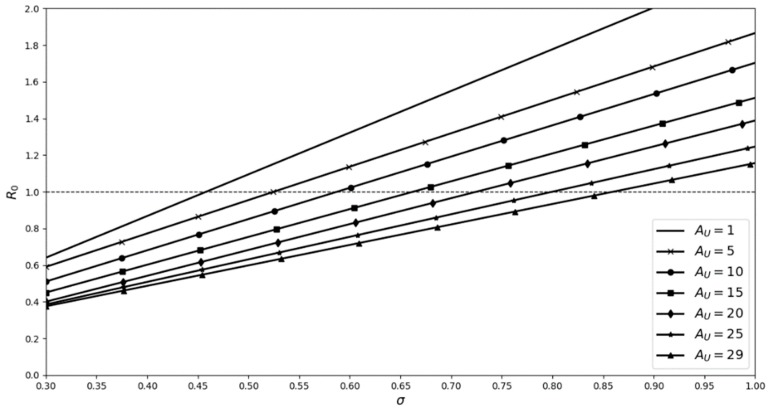
Ro values for the introduction of one A_H_ individual to populations with differing numbers of A_U_, while the total population size remains constant at 30. The number associated with each line indicates the number of A_U_ individuals present in the population. (M_D(U)_ = 0.775, Ψ = 0.45, M_N_ = 0.2) A_s_ is the number of susceptible individuals, Ψ is the contact rate, σ is the likelihood of transmission, M_N_ is the natural mortality rate, and M_D_ is the mortality rate associated with ranavirosis.

Increasing the number of A_U_ individuals in a population requires a higher transmission rate at each contact, for A_H_ to become established (i.e., R_o_ ≥ 1). However, establishment does not guarantee coexistence over the long term. While at first this might seem counter intuitive, it can be explained as follows—when there are more A_U_ individuals in the population there is a greater overall mortality rate because M_D(U)_ >> M_N_. Hence, there are actually fewer individuals to infect. Even when all of the population (*n* = 29) is composed of A_U_ individuals, A_H_ can become established (σ_3_ ≈ 0.85; see Figure 10). Although this is a high transmission rate, it is not unlikely in the situation where animals have broken skin (see [25]; adult common frogs with broken skin are more likely to become infected), which is characteristic of the ulcerative form of the ranaviral disease. The above analysis demonstrates that the two disease syndromes could persist in the short term, in the same population, by adult-to-adult transmission, albeit under conditions which have been estimated from data that are incomplete and not necessarily biologically relevant, based on the differing R_o_ values.

## 3. Discussion

Transmission dynamics are key to understanding host and pathogen persistence. In amphibian ranavirus systems, transmission can occur through direct contact, from scavenging, from virus particles persisting in the environment, and even between vertebrate classes, as seen in laboratory experiments. However, there is little understanding of the transmission routes that are most important in natural communities. In North America, ranavirus transmission appears to be primarily through direct contact with minimal transmission from water and scavenging, in most circumstances [16]. Our model is consistent with these results, demonstrating that the ranavirus(es) present in the UK common frog populations might persist in the short-term through horizontal, adult-to-adult transmissions alone.

When the declines of 8.1% per annum—observed by Teacher et al. [9] in the common frog populations where ranavirosis have emerged—are factored into the model, it took approximately 65 years for all adults in the population to become infected. This model did not take into account immigration, which has been noted by Teacher et al. [29] or the change in population structure that is caused by the emergence of ranavirosis, as described by Campbell et al. [30]. These additional factors, along with the effects of climate change [28], might change the outcomes of the models, and perhaps even require adjustments to the model parameters.

Strict adult-to-adult transmission of ranaviruses appears to be relatively rare. It is likely to have occurred in a mass mortality event of over 1000 adult and metamorphic water frogs (*Pelophylax* spp.) in the Netherlands, caused by a strain of *Common Midwife Toad Virus* (CMTV) [31]. In this case, there were also common newts (*Lissotriton vulgaris*) involved in the mortality event [31], but they made up only approximately 1% of the animals killed, and ranavirus transmission to the newts might be best attributed to pathogen spillover. In North America, reports of adult anurans infected with ranavirus are rare (e.g., only one adult wood frog [18]); morbidity and mortality events tend to occur in the tadpole stage. However, in the UK, adult-only mortality and morbidity events are typical, therefore, adult-to-adult transmission must play a major role in the transmission, which is consistent with our model results. This is an important finding since, there often is a lack of tadpoles in the ponds at the time of the adult mortality events [20]. However, other species might act as a reservoir of hosts that can infect common frogs [32], such as common toads, common newts (*Lissotriton vulgaris*) and the introduced common midwife toad (*Alytes obstetricans*). Future models and studies should explore interspecies transmission and subsequent population dynamics, especially since the presence of other species, namely common toads (*Bufo bufo*), can reduce disease risk in common frogs [5,27].

Our models showed that both disease syndromes can co-exist in the short term, despite competition between the ranavirus(es) associated with the different disease syndromes. This is not surprising if there are multiple strains of ranavirus present in the population. Exposure to different ranaviruses has been shown to result in enhanced viral infectivity in larval amphibians in the USA [33], and it is possible that this same pattern is occurring in the adult UK common frogs. The susceptibility of the host might depend on which ranavirus strain (ulcerative or hemorrhagic) is first introduced into the population. A similar effect has been previously observed in tadpoles exposed to *Frog virus 3* (FV3) and *Ambystoma tigrinum virus* [33]. This might be the case in the UK, because there are at least two different types of ranaviruses present [27]. CMTV-like and FV3-like ranaviruses have been previously identified in the UK populations of common frogs [27]; however, no association with the distinct disease syndromes were found in this study. In a previous study, molecular differences between two of the isolates (RUK 11 and RUK 13) were found, both of which were responsible for different disease syndromes. Duffus et al. [34] found that while the major capsid protein sequences for these two isolates were similar to FV3, the partial sequence of open reading frame 57r (an eIF-2α homologue) was similar to that found in an isolate of Chinese giant salamander virus (a common midwife toad-like virus). These differences could be indicative of larger scale molecular differences between the RUK isolates that result in the different ranaviral syndromes seen in common frogs in the UK.

Our models were greatly limited by a lack of robust parameter estimates. The contact rates were unknown for common frogs and the transmission coefficients were based on experiments with small sample sizes and unrealistic viral titres. Better parameter estimation (e.g., contact rates, individual susceptibility to ranavirosis, and disease-induced mortality in adults) would be key to improving the predictive values of all presented models.

## Figures and Tables

**Table 1:** Estimates for σ derived from the literature. Note: Experiments where the exposure was via inoculation have not been included in these estimates. No distinction has been made between the types of ranavirus-associated disease that the virus was derived from. Development of disease data, TCID_50_ and type of experiment information are summarized from Cunningham et al. [25]. (HS = Hemorrhagic and US = Ulcerative forms of ranavirosis.).

Development of Disease		Disease Prevalence	Type of Experiment/Exposure Type	Estimate of σ	TCID_50_
No. with Disease	Total No. Exposed				
3	20	15%	Immersion with virus from naturally disease tissue, with and without bacteria	0.15	10^1^/mL
9	20	45%	Immersion with virus from naturally disease tissue homogenate to animals with skin wounds, with and without bacteria	0.45	10^2^/mL HS 10^1.5^/mL US
9	10	90%	Immersion in virus from culture	0.90	10^2^/mL HS 10^1.5^/mL US
5	5	100%	Immersion in virus from virus culture to animals with wounded skin	1	10^5.6^ to 10^6.2^/mL
2	5	40%	Immersion in virus from tissue homogenate from naturally diseased animals to animals with wounded skin	0.40	10^3^/mL

**Table 2:** Estimates for σ derived from the literature, taking into account the different disease syndromes and type of syndrome that the virus was obtained from. U indicates the ulcerative form of the disease; H is the hemorrhagic form. The estimate of σ is simply the prevalence of the disease based on the presence of the signs of disease when the experiment terminated. The average estimate of σ is simply the mean of the estimates for each type of virus used for exposure. Development of disease data and type of experiment information are summarized from Tables 3–5 of Cunningham et al. [25].

Development of Disease				Type of Experiment/Exposure Type	Estimate of σ	Average Estimate of σ
No. with U	No. with H	No. with U & H	Total Exp.			
2	0	0	5	Immersion with virus from naturally disease tissue with bacteria (Ulcerative)	0.4	0.36
1	0	0	5	Immersion with virus from naturally disease tissue without bacteria (Ulcerative)	0.2	
2	0	0	5	Immersion with virus from naturally disease tissue to animals with skin wounds with bacteria (Ulcerative)	0.4	
0	0	0	5	Immersion with virus from naturally disease tissue to animals with skin wounds without bacteria (Ulcerative)	0	
2	2	0	5	Immersion in virus isolated from naturally diseased animals from virus culture (RUK 13, Ulcerative)	0.8	
0	0	0	5	Immersion with virus from naturally disease tissue without bacteria (Hemorrhagic)	0	0.44
0	0	0	5	Immersion with virus from naturally disease tissue with bacteria (Hemorrhagic)	0	
1	1	1	5	Immersion with virus from naturally disease tissue to animals with skin wounds with bacteria (Hemorrhagic)	0.6	
0	3	1	5	Immersion with virus from naturally disease tissue to animals with skin wounds without bacteria (Hemorrhagic)	0.8	
1	2	1	5	Immersion in virus isolated from naturally diseased animals from virus culture (RUK 11, Hemorrhagic)	0.8	
